# Protection of Oligodendrocytes Through Neuronal Overexpression of the Small GTPase Ras in Hyperoxia-Induced Neonatal Brain Injury

**DOI:** 10.3389/fneur.2018.00175

**Published:** 2018-03-21

**Authors:** Meray Serdar, Josephine Herz, Karina Kempe, Elke Winterhager, Holger Jastrow, Rolf Heumann, Ursula Felderhoff-Müser, Ivo Bendix

**Affiliations:** ^1^Department of Pediatrics I, Neonatology, University Hospital, University Duisburg-Essen, Essen, Germany; ^2^Imaging Center Essen, EM Unit, University Hospital Essen, University Duisburg-Essen, Essen, Germany; ^3^Institute of Anatomy, University Hospital Essen, University Duisburg-Essen, Essen, Germany; ^4^Biochemistry II, Molecular Neurobiochemistry, Faculty of Chemistry and Biochemistry, Ruhr University Bochum, Bochum, Germany

**Keywords:** preterm birth, brain injury, hyperoxia, neuronal Ras, white matter injury, neuroprotection

## Abstract

Prematurely born infants are highly susceptible to various environmental factors, such as inflammation, drug exposure, and also high environmental oxygen concentrations. Hyperoxia induces perinatal brain injury affecting white and gray matter development. It is well known that mitogen-activated protein kinase signaling is involved in cell survival, proliferation, and differentiation. Therefore, we aim to elucidate cell-specific responses of neuronal overexpression of the small GTPase Ras on hyperoxia-mediated brain injury. Six-day-old (P6) *syn*Ras mice (neuronal Ras overexpression under the synapsin promoter) or wild-type littermates were kept under hyperoxia (80% oxygen) or room air (21% oxygen) for 24 h. Apoptosis was analyzed by Western blot of cleaved Caspase-3 and neuronal and oligodendrocyte degeneration *via* immunohistochemistry. Short-term differentiation capacity of oligodendrocytes was assessed by quantification of myelin basic protein expression at P11. Long-lasting changes of hyperoxia-induced alteration of myelin structures were evaluated *via* transmission electron microscopy in young adult animals (P42). Western blot analysis of active Caspase-3 demonstrates a significant upregulation in wild-type littermates exposed to hyperoxia whereas *syn*Ras mice did not show any marked alteration of cleaved Caspase-3 protein levels. Immunohistochemistry revealed a protective effect of neuronal Ras overexpression on neuron and oligodendrocyte survival. Hyperoxia-induced hypomyelination in wild-type littermates was restored in *syn*Ras mice. These short-term protective effects through promotion of neuronal survival translated into long-lasting improvement of ultrastructural alterations of myelin sheaths in mice with neuronal overexpression of Ras compared with hyperoxic wild-type mice. Our data suggest that transgenic increase of neuronal Ras activity in the immature brain results in secondary protection of oligodendrocytes from hyperoxia-induced white matter brain injury.

## Introduction

Within the last decade, mortality of very preterm infants and critically ill-term born infants has decreased by 25% due to major advances in obstetrics and neonatal intensive care (1). However, perinatal brain injury is still a leading cause of death and disability in children (2). With approximately 5.5–11.4% of all live births, the number of prematurely born infants has increased in industrialized countries (3), which may further rise due to increasing infertility treatments, multiple pregnancies, and higher maternal age (4). Due to considerable progress in perinatal management of high-risk infants, long-term survival has become an almost expected outcome. However, even in the absence of severe intracranial pathology such as intraventricular hemorrhage or periventricular leukomalacia affecting 10% of very preterm born children (5), diffuse white matter (WM) injury and reduction of cortical gray matter volume are observed in most survivors. This is often associated with cognitive impairment and behavioral problems such as attention deficit disorder, autism, and development of psychiatric disease in later life (6–9).

Compared with intrauterine conditions, preterm infants suffer from chronic or fluctuating exposure to supra-physiological oxygen levels involved in prematurity-related diseases such as retinopathy of prematurity and bronchopulmonary dysplasia (10, 11). However, there is mounting evidence from clinical and experimental studies that large amounts of oxygen in the neonatal period also disturb brain maturation involving oligodendrocyte degeneration and impaired differentiation leading to hypomyelination (12–14). Importantly, these short-term alterations of myelination translate into long-lasting structural WM alterations (15–19) associated with hyperactivity and coordination deficits at adolescent age (20), and cognitive impairment persisting into adulthood (19, 21).

Several cellular mechanisms of hyperoxia-induced preterm brain injury including apoptosis, autophagy, differences in modulation of reactive oxygen species, and inflammation have been identified (17, 21–26). At the molecular level, our previous work demonstrated the importance of the mitogen-activated protein kinase (MAPK) signaling pathway (22, 27). It was shown that hyperoxia reduces Ras GTPase activity and downstream signaling pathways (e.g., phosphorylation of the extracellular regulated kinase (ERK)) (27), which modulate important cellular responses such as differentiation and survival (28). Of note, using *syn*Ras-transgenic mice overexpressing constitutively activated Ras (29) in the neuronal compartment, we confirmed the important involvement of this pathway demonstrating protection against oxygen-induced cell death (27). However, long-term consequences for the development of WM injury and direct effects on hyperoxia-induced oligodendrocyte degeneration and hypomyelination are still unknown. Moreover, neuronal Ras activation in adult mice leads to an increase of dendritic spine density and synapses as well as increased neuronal activity (30). Taking into account that oligodendrocyte development is not only controlled intrinsically, e.g., by transcription factors but also by neuronal activity (31–33), modulation of neuronal activity and signaling pathways may determine oligodendrocyte responses and subsequent effects on myelination in oxygen-related injury of the immature brain.

Although our understanding of consequences of neonatal hyperoxia on WM development significantly improved within the last decade, the impact of a potential cross talk between the neuronal compartment and myelinating oligodendrocytes is still unknown. Given the important role of MAPK signaling in oxygen-induced preterm brain injury and the fact that neuronal activity modulates oligodendrocyte development, we aimed to elucidate the impact of a transgenic overexpression of constitutively activated Ras in neurons on hyperoxia-induced oligodendrocyte degeneration, myelination, and long-lasting ultrastructural alterations of the WM.

## Materials and Methods

### Animals and Housing

Transgenic mice overexpressing the mutated human-Ras gene [Ha-Ras G12V (exchange of glycin through valin)] under the synapsin 1 promoter (*syn*Ras) were used. These mice reveal a constitutive activation of Ras in post-mitotic neurons starting at postnatal day 4 (P4). Transgenic males were mated with wild-type female C57BL/6 mice. Genotyping was performed by PCR as described previously (29). The following primers were used to identify the mutated human transgene G12V (360 bp); Fwd (*syn*Ras for) AAT TCC AGC TGA GCG CCG GT and Rev (*syn*Ras rev) GAC ACA CTC ATG AGA TGC CT. Animals were housed under 12-h light/dark cycle, food and water *ad libitum*. All animal experiments were approved and performed in accordance with the guidelines of the University Hospital Essen, Germany and with local government approval by the State Agency for Nature, Environment and Consumer Protection North Rhine-Westphalia.

### Experimental Procedures

Due to the well-known delay in the brain growth spurt period, which corresponds to the most susceptible period of brain injury in neonatal rodents (34–36), 6-day-old mice were used. *Syn*Ras pups and their appropriate wild-type littermates were placed in an oxygen chamber containing 80% oxygen (OxyCycler, BioSpherix, USA) for 24 h (HO) together with their lactating dams. Control pups were kept under normoxic conditions (21% oxygen, NO) resulting in four study groups: wild-type hyperoxia (BL6 HO); *syn*Ras hyperoxia (*syn*Ras HO); wild-type normoxia (BL6 NO); and *syn*Ras normoxia (*syn*Ras NO). Pups were sacrificed at P7, P11, and P42 under deep anesthesia. In accordance with our previous studies (17, 21, 27, 37), 24 h is sufficient to modulate myelin basic protein (MBP) expression and acute cellular degeneration determined at P11 and P7, respectively, without an induction of acute injury to the dams (27, 37). Since differences in MBP expression following neonatal hyperoxia are less prominent few days after the peak of brain growth spurt period (P7–P10) initial myelination was assessed at term-equivalent age (P11) (34, 36) according to our previous work (15, 17, 19, 21, 37). Ultrastructural alterations of myelination were analyzed in young adult mice at P42 by transmission electron microscopy as described below. For protein analysis, mice were transcardially perfused with PBS and brain hemispheres were snap frozen in liquid nitrogen. For histological studies, pups were transcardially perfused with PBS followed by 4% paraformaldehyde (PFA, Sigma–Aldrich). Brains were postfixed in 4% PFA overnight at 4°C and embedded in paraffin. Based on our previous experience (17, 21), whole hemispheres (excluding cerebellum) were used for Western blot analysis of MBP and cleaved Caspase-3 expression.

### Immunoblotting

Western blotting was performed as described previously (17, 21), with adaptions in epitope detection. Membranes were blocked with 5% non-fat dry milk in Tris-buffered saline, 0.1% Tween-20 (TBST, Sigma Aldrich) and incubated overnight (4°C) with the following primary antibodies: polyclonal rabbit anti cleaved Caspase-3 (1:1,000, Cell Signalling, Germany), monoclonal mouse anti-MBP antibody (1:10,000, Abcam, UK), monoclonal mouse anti-Olig2 (1:1000, Millipore, Germany), and polyclonal rabbit anti-glyceraldehyde 3-phosphate dehydrogenase antibody (1:1,000, Santa Cruz, Germany). Horseradish peroxidase-conjugated secondary anti-mouse (1:5,000) or anti-rabbit (1:2,000, both DAKO, Denmark) antibodies were used. All antibodies were diluted in 5% non-fat dry milk in TBST. Antibody binding was detected by using enhanced chemiluminescence (GE Healthcare Life Sciences, Germany). For visualization and densitometric analysis, ChemiDoc™ XRS+ imaging system and ImageLab software (Bio-Rad, Germany) were used. Representative images of full-length Western blot are depicted in Figure S1 in Supplementary Material.

### Immunohistochemistry and Confocal Microscopy

After deparaffinization, 10 µm coronal sections (-2.255 ± 0.6 mm from bregma) were rehydrated. Antigen retrieval was performed in a preheated 10 mM sodium citrate buffer (pH 6.0) for 30 min. After blocking with 1% bovine serum albumin, 0.3% cold fish skin gelatine in 0.1% Tween-20 TBS (all Sigma–Aldrich) slides was incubated with primary antibodies overnight at 4°C followed by appropriate secondary antibody incubation for 1 h at room temperature. Sections were counterstained with 4,6-diamidino-2-phenylindole (DAPI) (1 µg/ml, Invitrogen, Germany). Myelination was evaluated at P11 using the primary mouse anti-rat MBP antibody (1:100, SMI-99, Sternberger Monoclonals, USA). Degeneration of oligodendrocytes or neurons were detected at P7 *via* co-labeling of Olig2 (1:100, monoclonal mouse anti-Olig2, Millipore) or NeuN (1:200, polyclonal rabbit anti-NeuN, Millipore) with terminal deoxynucleotidyl transferase mediated biotinylated dUTP nick end labeling (TUNEL, *In Situ* cell death detection kit, FITC; Roche, Germany), performed according to the manufacturers’ instructions. All primary antibodies were followed by appropriate secondary antibody staining (anti-mouse Alexa Fluor 488, anti-rabbit Alexa Fluor 555, anti-mouse Alexa Fluor 555; all 1:500, Invitrogen). Stained brain sections were analyzed by confocal microscopy (A1plus, Eclipse Ti, with NIS Elements AR software, Nikon, Germany) using 10× or 20× objectives. Three laser lines (laser diode: 405 nm; Ar laser: 514 nm; G-HeNe laser: 543 nm) and three different filters (450/50-405 LP, 515/20-540 LP, 585/65-640 LP) were used for image acquisition. Confocal *z*-stacks of 10 µm thickness (*z*-plane distance 3 µm) were converted into two-dimensional images using maximum intensity projections. Image acquisition and analysis were performed by an observer blinded to treatment and genotype. The quantification of each staining was performed with the NIS Elements AR software. For MBP stained sections, large scale images (stitching) of deep cortical WM were generated. Degenerating oligodendrocytes or neurons were quantified by counting triple positive cells (DAPI, TUNEL, Olig2/NeuN) in eight bilateral regions of interest (ROI) derived from two sections per animal (each ROI: 396,900 µm^2^, retrosplenial and primary somatosensory cortex; cingulum, deep cortical WM; parafascicular nucleus and posterior complex of the thalamus; CA1 and CA2 of the hippocampus).

### Electron Microscopy

Six-week-old transgenic *syn*Ras mice and their appropriate wild-type littermates exposed to 24 h hyperoxia at P6 were perfused with 37°C warm phosphate buffer solution followed by 2.5% glutaraldehyde in 0.1 M cacodylate buffer (CB). Brains were removed and immediately transferred into 12-well plates filled with 2.5% glutaraldehyde in 0.1 M CB. Brains were cut into 1 mm thick frontal slices for better penetration. One section clearly showing the corpus callosum was chosen from each specimen and trimmed in a way that the latter was preserved in a final ~1 mm × 1 mm × 1 mm tissue block. After fixation lasting for at least 12 h, blocks were washed in CB at room temperature for 3× 30 min, followed by 1% osmium tetroxide (PolyScience, USA) in CB for 3.5 h in the dark. After washing in CB, 30 and 50% ethanol (45 min each) was added followed by incubation in 1% uranylacetate (Electron Microscopy Science, USA) in 70% ethanol in the dark for 1 h. Blocks were further incubated in 80, 90, and 96% ethanol (45 min each) and 100% ethanol (3 × 20 min) followed by propylene oxide (2× 20 min, Sigma) and EPON^®^ (Sigma) solutions in propylene oxide with increasing EPON^®^ concentration (3:1, 1:1, 1:3; 60 min each, 100% EPON^®^ overnight) at room temperature. Polymerization was performed at 60°C for 2 days. After trimming solid EPON^®^ blocks were cut on a Reichert-Jung Ultracut^®^ E ultramicrotome (Reichert AG, Austria) set to a thickness of 50 nm. Sections were mounted on 200 mesh hexagonal copper grids and treated with 1% uranylacetate for 4 min and lead citrate trihydrate (0.4% in ddH_2_O) for 3 min for contrast enhancement. A Zeiss transmission electron microscope (EM 902A; Zeiss, Germany) was used for final investigation at 80 kV at magnifications from 3,000× to 100,000×. Digital image acquisition was performed by a Mega View III slow-scan-CCD camera connected to a PC running ITEM^®^ 5.0 software (Soft Imaging Systems, Germany); images were saved as uncompressed 16 bit TIFF files and further processed using Adobe^®^ Photoshop^®^ CS5. Alterations of the myelin sheath were analyzed by using the Image J software (Java Software).

To evaluate myelin deficits a total of 1,645 axons (BL6 HO) and 1,488 axons (*syn*Ras HO) in 60–65 fields of view at 10,000× magnification (61.44 µm^2^ each) derived from 4 mice per group were analyzed. The percentage of non-myelinated axons related to the total number of axons per field of view was quantified. Pathological alterations in the myelin sheath of myelinated axons were evaluated through quantification of the number of axons with myelin encapsulation and decompaction, and axons with increased adaxonal space. Only axons whose contour was completely within each photograph were used. Analysis was carried out by an individual blinded to group assignment.

### Statistical Analysis

**Figure 1:**
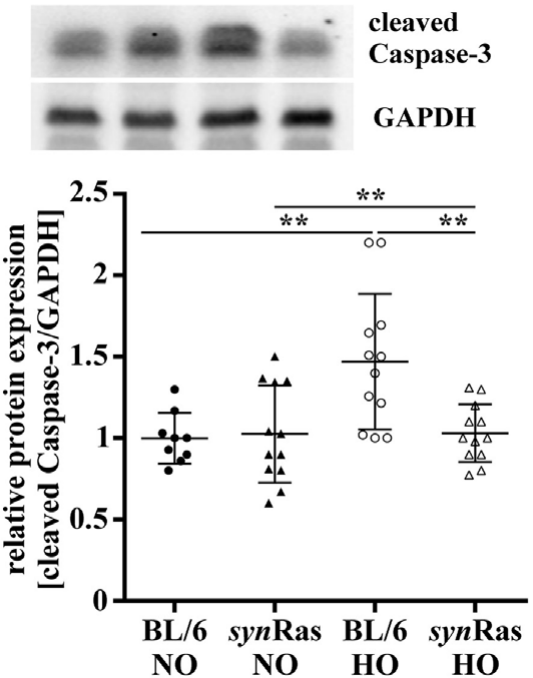
Apoptosis caused by hyperoxia is reduced in transgenic *syn*Ras mice. Induction of apoptosis was determined by relative protein expression analysis for cleaved Caspase-3 by Western blot in lysates from hemispheres of postnatal day 7 (P7) mice that were exposed to either normoxia [21% oxygen (NO)] or hyperoxia [24 h, 80% oxygen (HO)] at P6 in *syn*Ras mice or their respective wild-type littermates (BL/6). Data are represented as relative protein expression [cleaved Caspase-3/glyceraldehyde 3-phosphate dehydrogenase (GAPDH)] normalized to the control group (BL/6 NO) (**p < 0.01).

**Figure 2:**
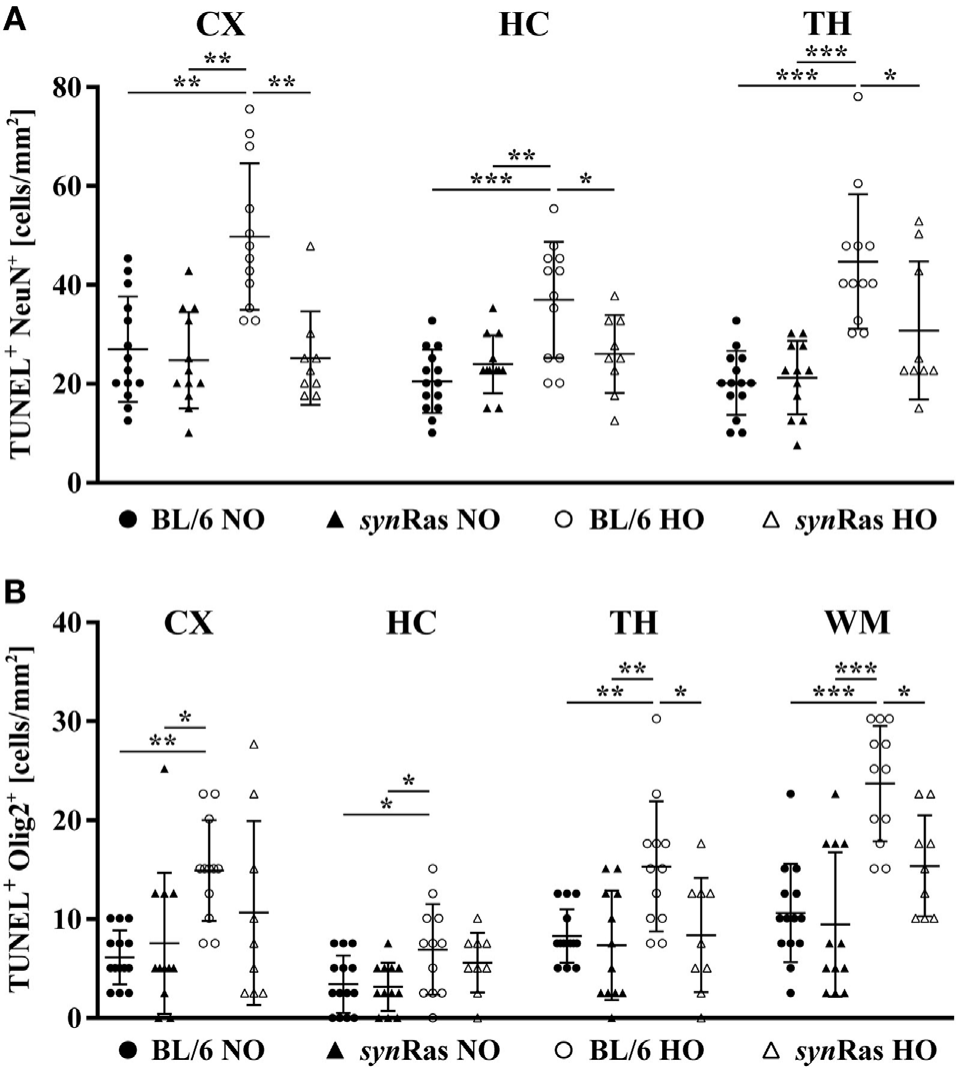
Hyperoxia-induced neuronal and oligodendrocyte degeneration is ameliorated in *syn*Ras mice. Brain sections from P7 mice either exposed to normoxia [21% oxygen (NO)] or hyperoxia [24 h, 80% oxygen (HO)] at P6 in *syn*Ras mice or their respective wild-type littermates (BL/6) were analyzed. Neuronal (NeuN) **(A)** and oligodendrocyte (Olig2) **(B)** degeneration was determined in cortical (CX), hippocampal (HC), thalamic (TH), and white matter (WM) regions by double-labeling with TUNEL (*p < 0.05, **p < 0.01, and ***p < 0.001).

**Figure 3:**
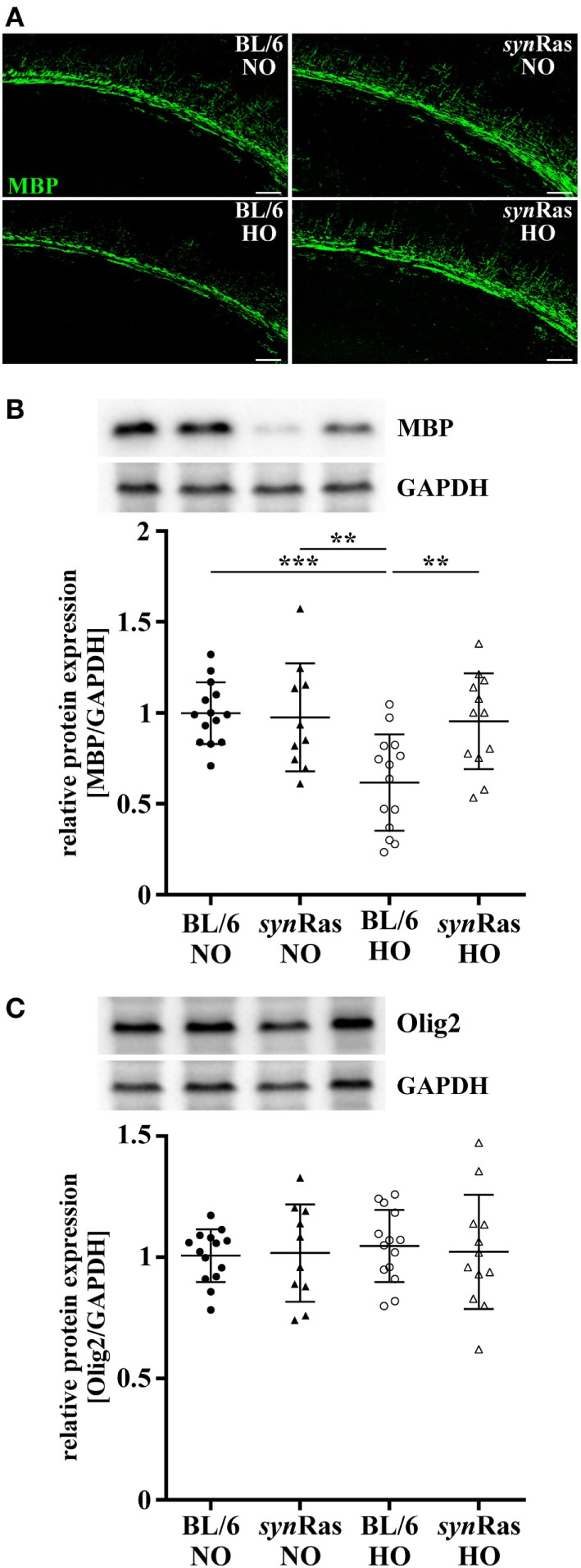
Impact of neuronal Ras activation in *syn*Ras on short-term hyperoxia-induced hypomyelination. Myelin basic protein (MBP) expression was analyzed 4 days post hyperoxia. **(A)** Representative immunohistochemical staining of the deep cortical white matter assessed by confocal microscopy (scale bar = 100 µm). **(B)** MBP and **(C)** Olig2 protein expression was analyzed by Western blot in protein lysates of hemispheres of P11 mice that were exposed to either normoxia [21% oxygen (NO)] or hyperoxia [24 h, 80% oxygen (HO)] at P6 in *syn*Ras mice or their respective wild-type littermates (BL/6). Data are represented as relative protein expression [MBP/glyceraldehyde 3-phosphate dehydrogenase (GAPDH) and Olig2/GAPDH] normalized to the control group (BL/6 NO) (**p < 0.01 and ***p < 0.001).

**Figure 4:**
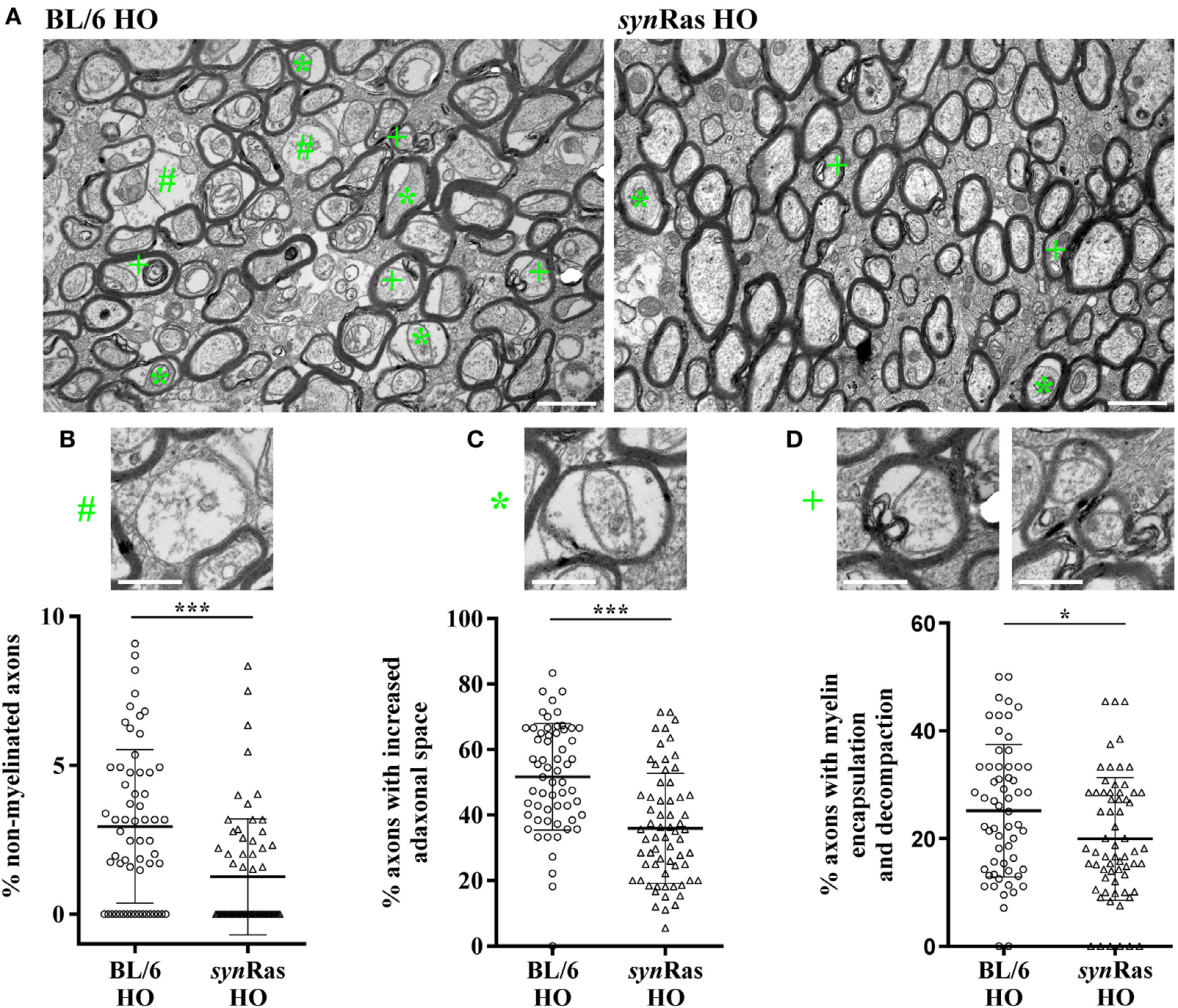
Long-lasting ultrastructural changes of myelination in hyperoxic animals are diminished in transgenic *syn*Ras mice. **(A)** Representative electron microscopy images of young adult (P42) BL/6 and *syn*Ras mice either exposed to normoxia [21% oxygen (NO)] or hyperoxia [24 h, 80% oxygen (HO)] at P6. Quantification of non-myelinated axons [**(B)**, #], increased adaxonal space [**(C)**, *], and axons with encapsulated and decompacted myelin sheets [**(D)**, +]. Alterations are exemplified in panel **(A)**. Scale bar in panel **(A)** is 1 and 0.5 µm in higher magnified images in panels **(B–D)**. *n* = 60–65 analyzed field of views (61.44 µm^2^ each) derived from four mice per group (*p < 0.05 and ***p < 0.001).

Data are presented as mean + SD. For statistical analysis, the GraphPad Prism 6.0 software package (GraphPad Software, USA) was used. After checking for normal distribution using D’Agostino–Pearson omnibus test either ordinal one-way analysis of variance with *post hoc* Bonferroni multiple comparisons test or Kruskal–Wallis with Dunn’s multiple comparisons test were applied (Figures 1–3). For comparison of two groups (Figure 4) either Student’s *t*-test (parametric) or in case of non-parametric data Mann–Whitney *U* test was used. *p*-Values less than 0.05 were considered as statistically significant.

## Results

### Neonatal Hyperoxia-Induced Apoptosis Is Decreased by Neuronal Ras Activation

To evaluate the extent of brain injury following neonatal hyperoxia at P6, we determined cleaved Caspase-3 protein abundance immediately after 24 h hyperoxia. In line with previous findings (27), we detected a significant increase of cleaved Caspase-3 levels in protein lysates of wild-type mice following hyperoxia (BL/6 HO) compared with normoxic controls (BL/6 HO) and compared with *syn*Ras mice exposed to hyperoxia (*syn*Ras HO) (Figure 1). No alteration of cleaved Caspase-3 protein levels was determined in *syn*Ras animals at normoxia (*syn*Ras NO, Figure 1).

### Constitutive Neuronal Ras Activity Ameliorates Neuronal and Oligodendrocyte Degeneration in Neonatal Mice Following Hyperoxia

To investigate the effect of hyperoxia and neuronal Ras overexpression at a cellular level, indicated by TUNEL labeling, we analyzed the amount of degenerating neurons (Figure 2A) and oligodendrocytes (Figure 2B). Hyperoxia significantly increased neuronal degeneration in cortical, hippocampal, and thalamic (TH) regions of wild-type mice, which was significantly reduced in *syn*Ras-transgenic mice (Figure 2A). For oligodendrocyte degeneration, we detected a similar increase in BL/6 wild-type mice (Figure 2B). Importantly, selective neuronal activation of Ras led to a significant decrease of oligodendrocyte degeneration in TH and WM structures (Figure 2B).

### Hyperoxia-Induced Hypomyelination Is Prevented in *syn*Ras Mice

A major hallmark of oxygen-induced toxicity to the immature brain is characterized by hypomyelination (15, 19, 21). To clarify whether the unexpected decrease in oligodendrocyte degeneration in *syn*Ras mice translates into disturbed myelination, we next examined MBP expression at P11 after exposure to hyperoxia at P6. As shown in representative images in Figure 3A, hyperoxia resulted in a decrease of MBP expression in wild-type BL/6 mice which was restored in *syn*Ras mice (Figure 3A). Quantification of protein lysates by Western blot analysis isolated from whole hemispheres confirmed these qualitative observations and revealed significantly reduced levels of MBP expression in animals exposed to hyperoxia compared with normoxic control wild-type control mice (Figure 3B). Normoxic and hyperoxic *syn*Ras mice revealed similar levels of MBP expression compared with normoxic BL/6 controls (Figure 3B). Of note, 4 days after hyperoxia Olig2 expression was not significantly altered across all experimental groups (Figure 3C).

### Neuronal Ras Activation Protects From Hyperoxia-Induced Long-Lasting Ultrastructural Myelination Abnormalities

Since our major focus of this study was to determine the impact of enhanced neuronal Ras activity in the context of hyperoxia-induced WM pathology and according to the fact that we did not observe any differences between normoxic control animals (BL/6 and *syn*Ras NO) across all previous analyses, we focused our analysis regarding long-term ultrastructural changes *via* electron microscopy to hyperoxic animals only. Assessing cross-sections of the corpus callosum of 6-week-old mice after neonatal hyperoxia, we observed several myelin abnormalities including non-myelinated axons, axons with an increased adaxonal space as well as split sheaths with decompaction, myelin lamellae partially broken down into vesicular structures at the innermost region of the myelin sheath (internal loops) as well as focal myelin damage (Figure 4A). Comparing both injury groups, we detected a significant reduction regarding non-myelinated axons (Figure 4B), axons with an increased adaxonal space (Figure 4C), as well axons with signs of myelin encapsulation and decompaction (Figure 4D) in *syn*Ras following neonatal hyperoxia at P6.

## Discussion

Oxygen-induced preterm birth-related brain injury is associated with subtle neurodegeneration and impaired WM development resulting in long-lasting decrease of fractional anisotropy and diffusivity in the WM associated with neurodevelopmental disturbances (17, 19–21, 38, 39). Myelination is the key for efficient nerve conduction velocity proposed to strengthen circuitry throughout the nervous system (40). However, recent evidence also suggests a substantial impact of neuronal activity on myelination (31, 33). Our results demonstrate that the selective neuronal activation of the small GTPase Ras protects against hyperoxia-mediated neuronal but also oligodendrocyte degeneration and hypomyelination. Importantly, these short-term effects translated into long-lasting improvement of myelin integrity resulting in a reduced amount of non-myelinated axons, an increase in axon-lamination, and an elevated number of axons with compact myelin.

Small GTPases from the Ras family are highly evolutionary conserved, molecular switches involved in important cellular responses such as proliferation, differentiation, and survival (28). We previously showed that hyperoxia reduces Ras activity and that transgenic mice overexpressing Ras under the synapsin 1 promoter (*syn*Ras) to drive the selective overexpression of V12-Ha-Ras in post-mitotic neurons (29) are protected against hyperoxia-induced cellular degeneration (27). Nevertheless, the cellular target of protection remained unclear. In this study, we show that cell-specific neuronal modulation of Ras protects neurons from cellular degeneration. According to our previous work demonstrating that hyperoxia-induced cell death is associated with reduced expression Ras effector molecules promoting cell survival (e.g., ERK1/2) (27), results of this study can be most likely explained by increased Ras-mediated neuronal survival signaling in *syn*Ras mice. Our results of regional neuronal cell death analysis correspond to previous studies revealing increased cell death in the cortex and the deep gray matter following hyperoxia (41, 42). Of note, we also detected an increased neuronal cell death in parts of the hippocampus (i.e., CA1 and CA2) which seems to contrast a previous report revealing no impact of hyperoxia on cellular degeneration in the dentate gyrus (42). This difference might be explained by the different substructures analyzed in the former study and by the fact that the dentate gyrus belongs to the archicortex which respond different to cellular stress compared with the neocortex (43). Furthermore, in contrast to the previous study, we performed cell-specific analysis by NeuN/TUNEL co-staining to specifically analyze neuronal degeneration which is limited with a single TUNEL staining. Even though the importance of Ras in cell survival is widely accepted, for specific pathophysiological cases and cellular systems, it has been shown that Ras may also promote cell death (44). In this study, there are no indications for such detrimental effects probably due to developmental differences, i.e., transgenic *syn*Ras mice exposed to normoxia did not show an increased cellular degeneration compared with wild-type littermate controls. Therefore, the immature brain might be less sensitive to proapoptotic effects of Ras activation and thus potential harmful effects.

Surprisingly, in addition to protection from neuronal death, we observed a marked decrease in hyperoxia-induced oligodendrocyte degeneration in *syn*Ras-transgenic mice. Since in these mice, Ras activity is only enhanced in neurons, an indirect impact of the neuronal compartment on oligodendrocyte responses to hyperoxia can be assumed. These results clearly support the current concept of an intense communication between neurons/axons and oligodendrocytes/myelin (31, 33). Interestingly, elevated Ras signaling exclusively in oligodendrocytes has been shown to mediate opposite effects (45). Increased Ras activity in oligodendrocytes of healthy adult mice resulted in myelin decompaction (45) emphasizing the importance of cell-specific analysis and the divergent roles of Ras-mediated signaling dependent on the pathophysiological context, the cellular system, and developmental stage. Nevertheless, expression was restricted to mature oligodendrocytes. Novel transgenic mouse models directly targeting immature oligodendrocyte precursor cells (OPCs) would help to clearly define the interplay between neuronal responses to hyperoxia and developmental processes of myelination in neonatal subjects.

Protective effects on acute oligodendrocyte degeneration were accompanied by improved myelination 4 days after hyperoxia. Interestingly, no differences were observed for the abundance of Olig2 at this time point, indicating compensatory oligodendrocyte proliferation, which, however fails to restore myelination deficits (37). This might be well explained by impaired oligodendrocyte maturation and differentiation following hyperoxia as previously described (21, 37). Increased MBP expression at similar Olig2 levels in hyperoxic *syn*Ras suggests that neuronal Ras activation positively influences differentiation capacity of OPCs in the context of neonatal oxygen-induced toxicity.

In addition to subacute effects on oligodendrocyte survival and differentiation, neonatal hyperoxia causes long-lasting structural alterations on tensor imaging scans of adult brains, which is accompanied by significant cognitive deficits (21). In this study, we provide more detailed information about axon–myelin integrity with ultrastructural analysis of myelin sheaths in young adult animals exposed to neonatal hyperoxia. Here, we detected a considerable amount of non-myelinated axons in hyperoxic wild-type mice, which was significantly improved in *syn*Ras mice. Furthermore, the percentage of myelinated axons with abnormal myelin structures was significantly lower in *syn*Ras mice. While being in concordance with previous descriptions of hyperoxia-related disturbed ultrastructural myelin integrity (18), our results add important new knowledge, because pathological alterations of the WM were improved solely by modulation of the neuronal compartment, represented by increased Ras activity.

Different mechanisms may account for our observations. First, according to the initial description by Heumann et al. *syn*Ras mice reveal an increased expression of neuropeptide Y (29), which has been shown to improve myelination in the neonatal brain *via* induction of neurotrophin 3 (46). Second, increased neuronal activity described for *syn*Ras mice displayed by enhanced glutamatergic transmission, and long-term potentiation (30) may explain our findings. Whereas the essential need of intact myelination for preservation and maintenance of axonal structure and function is without any doubt (47), there is compelling evidence for a substantial communication into the other direction, i.e., an activity-dependent signaling from neurons/axons to oligodendrocytes/myelin (33). Accordingly, recent studies indicate that axonal action potentials activate myelinic NMDA receptors (48) resulting in impaired metabolic coupling between axons and oligodendrocytes (49). In addition to metabolic processes, neuronal activity is supposed to determine the release of BDNF (50), a growth factor particularly important for OPC development and maturation (51, 52). While much focus has been given to neuronal activity affecting OPC responses during physiological development, potential interactions in response to pathology in the developing brain are less explored. Considering the aforementioned studies, we speculate that hyperoxia modulates neuronal/axonal function and activity due to reduced Ras activity, which may be compensated by neuronal Ras overactivation thereby improving oligodendrocyte differentiation and long-term myelination capacity. A clear goal for future work will be to characterize neuronal/axonal function and activity in hyperoxic animals with constitutively expressed activated Ras in the neuronal compartment. This is further supported by a very recent report that the pattern of neuronal activity triggers distinct responses of OPC proliferation and differentiation (53).

Strengths of this study are well statistically powered analyses, long-term evaluation of myelination deficits, and the use of a cell-specific transgenic mouse model. A potential limitation might be that this mouse model did not allow for conditional transgene expression initiated at various time points. Nevertheless, as in *syn*Ras mice, constitutive Ras activation starts at postnatal day 4, physiological effects on embryonic neuronal development can be excluded. Furthermore, long-term analyses of neurodevelopmental behavior and of axonal function/integrity would have strengthened our hypothesis. However, previous experimental studies combined with clinical data provide clear evidence for a good correlation between alterations of WM development and long-term behavioral deficits as well as axonal integrity and function (14, 18–21, 39).

To conclude, this work demonstrated that hyperoxia-induced impairment of neurodevelopment does not solely rely on direct modulation of oligodendrocyte responses but is also affected by neuronal cell signaling with major impact on WM development. This work emphasizes the unmet need for cell-specific analysis in models of neonatal brain injury to identify more specific targets for therapeutic intervention.

## Ethics Statement

All animal experiments were approved and performed in accordance with the guidelines of the University Hospital Essen, Germany and with local government approval by the State Agency for Nature, Environment and Consumer Protection North Rhine-Westphalia.

## Author Contributions

MS, IB, JH, KK, RH, and HJ designed and performed the experiments and analyzed the data. MS, JH, RH, EW, HJ, UF-M, and IB discussed the data. UF-M and IB initiated and organized the study. MS, JH, UF-M, and IB wrote the manuscript.

## Conflict of Interest Statement

The authors declare that the research was conducted in the absence of any commercial or financial relationships that could be construed as a potential conflict of interest.
