# Supplementary material for: Protection of Oligodendrocytes Through Neuronal Overexpression of the Small GTPase Ras in Hyperoxia-Induced Neonatal Brain Injury
Source: Front Neurol. 2018 Mar 21;9:175. doi: 10.3389/fneur.2018.00175 (PMC5871665; doi:10.3389/fneur.2018.00175)
Supplement: Supplementary file 1 [file Image_1.PDF]

Supplementary figure

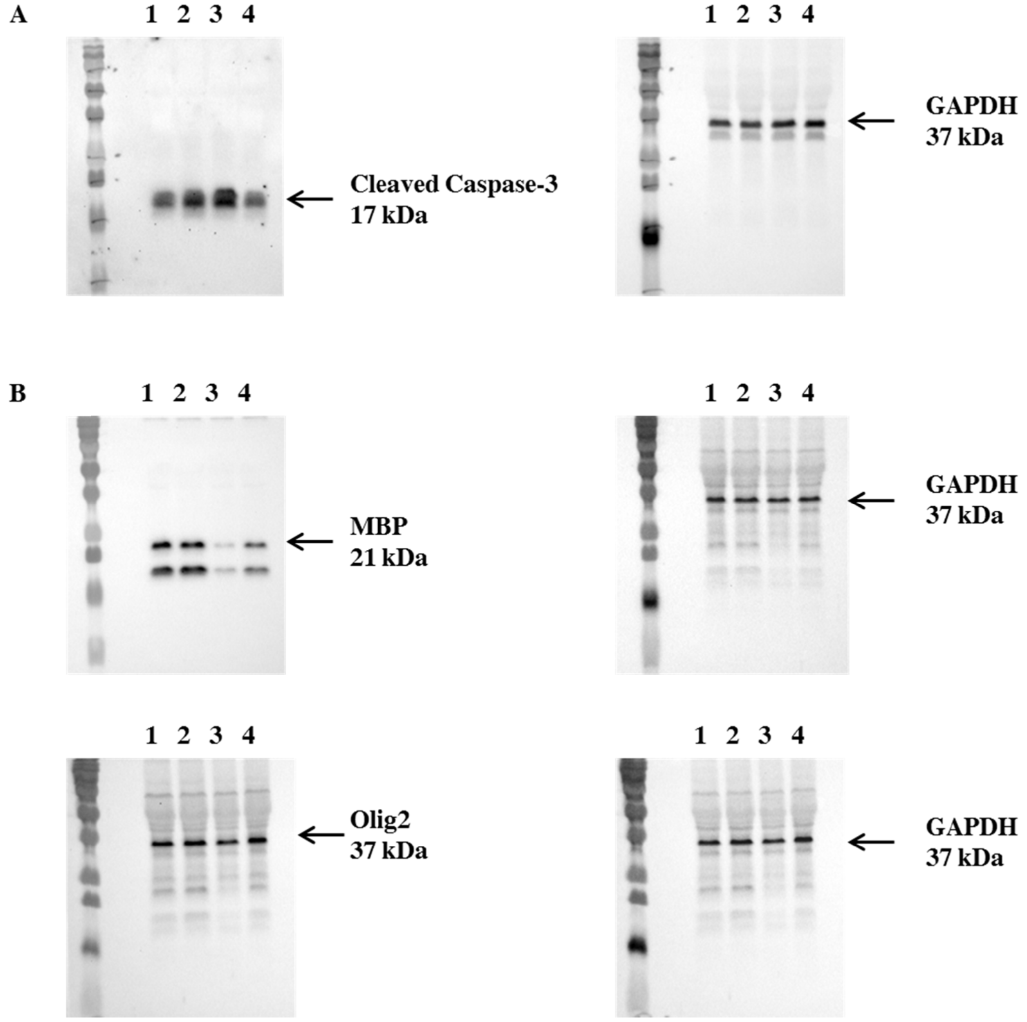

Representative full-length Western blot images.

A) Representative images of cCaspase-3 and the reference protein GAPDH. B) Images of full-length membranes of MBP (upper panel) and Olig2 (lower panel) with their corresponding GAPDH images, 1, BL6/ NO; 2, *syn*Ras NO; 3, BL6/HO; 4, *syn*Ras HO.
